# Is It Time to Step outside the Laboratory? The Feasibility of Field-Based Examination of Exercise-Induced Hypoalgesia in Elite Badminton Athletes with and without Knee Pain

**DOI:** 10.1155/2024/2953220

**Published:** 2024-06-11

**Authors:** Brooke K. Coombes, M. Dilani Mendis, Felix Leung, Julie A. Hides

**Affiliations:** ^1^School of Health Sciences and Social Work, Griffith University, Nathan Campus, Nathan, QLD 4111, Australia; ^2^Menzies Health Institute Queensland, Griffith University, Nathan Campus, Nathan, QLD 4111, Australia

## Abstract

**Aim:**

To investigate the feasibility of testing exercise-induced hypoalgesia (EIH) in a field setting. The effect of knee pain on EIH was also explored.

**Design:**

Within-group pre-post design.

**Materials and Methods:**

Fourteen athletes (8 male, 6 female) competing at an international level in badminton were tested on the sideline during an in-season training session. Participants completed questionnaires and a single leg decline squat to evaluate the presence of knee pain. A blinded examiner measured PPT over the quadriceps muscle before and after two conditions (3-minute quiet rest and 3-minute isometric wall squat).

**Results:**

The exercise protocol was completed by 13 (93%) participants. Mean (SD) exertion was 8.4 (1.7), and mean thigh pain was 7.9 (2.0) at 3 minutes. Very high reliability was observed for PPT collected before and after rest (ICC 0.94, 95% CI 0.85, 0.98). PPT significantly increased by 22.4% (95% CI 15.1, 29.7) after wall squat but not after rest. Relative increases in PPT were similar in participants with and without knee pain on single leg decline squat (22.2% versus 22.6%, 7 participants each).

**Conclusion:**

Simple, field-based tests of endogenous analgesia are feasible and could provide new opportunities to evaluate an athlete's risk of persistent pain.

## 1. Introduction

Overuse injuries of the knee are a common finding in badminton athletes. Globally, the incidence of injury among elite badminton players ranges between 1.6 and 2.9 per 1000 playing hours [1]. A recent study of 500 able-bodied Malaysian, elite badminton players found that the knee was the most prevalent region of injury, with the three most frequently diagnosed problems being patellar tendinopathy (23%), ligament and meniscus injuries (21%), and patellofemoral pain syndrome (13%) [1]. Most injuries occurred during training with overuse being the most common mechanism (39%). Knee injuries may directly affect performance in badminton, as athletes must perform quick changes of direction, jumps, and lunges at the net [2]. Furthermore, incomplete recovery and residual symptoms may also expose players to the development of chronic pain.

Exercise is a core component in the treatment of both patellar tendinopathy and patellofemoral pain [3, 4]. Since pain is considered as one of the major barriers to exercise adherence, it is important to understand the effects of acute exercise on endogenous analgesia mechanisms [5]. In healthy individuals, an acute bout of exercise (e.g., an isometric wall squat) is associated with increased pressure pain thresholds (PPTs) over exercising and nonexercising muscles, often with a duration lasting from 5 to 30 minutes after exercise [6]. This effect is termed exercise-induced hypoalgesia (EIH). The magnitude of the EIH response is typically calculated as the absolute or relative difference in the test stimulus (e.g., PPT) after the exercise condition compared with before the exercise condition [6]. Impairments in EIH have been observed in people with various chronic musculoskeletal conditions, including those of the knee [7, 8]. To date, there are limited studies in athletic participants [9], particularly those that engage in dynamic sports such as badminton where musculoskeletal pain may limit an athlete's participation in sport.

The current study explored the hypoalgesic effects of an acute bout of isometric exercise in a field setting in elite badminton athletes with and without knee pain. A within-group pre-post design was used to compare PPT of the quadriceps muscles after quiet rest and wall squat exercise. Our primary aim was to investigate the feasibility of the protocol within the proposed setting. We hypothesised that it would be feasible for athletes to complete a 3-minute wall squat and that PPT would be reliable when tested within the field setting. Our secondary aim was to investigate whether knee pain observed during a standardised clinical test influenced the magnitude of EIH, providing sample size calculations for future study.

## 2. Materials and Methods

Participants included male and female athletes aged 18 years and over, who had represented Australia in badminton at an international level. Athletes available during a routine training session at the Melbourne Sports and Aquatic Centre (MSAC, Melbourne, Australia) over a two-day period in April 2023 consented to participate. There were no exclusion criteria. Ethical approval was received from Griffith University (2017/896), and approval for testing was received from Badminton Australia. All work was conducted in accordance with the Declaration of Helsinki (1964). Participants were provided with an information sheet prior to the training session, and athletes provided written, signed consent on the day of testing. All testing was completed over 2 days on the sideline within their usual 3-hour early morning training session. Badminton training consisted of game drills, and the same training activities were performed on both testing days. Coaches allocated participants to testing on one of the two days to limit disruption to the team's training. Participants were asked to rate the intensity of training completed prior to experimental testing on the OMNI-Resistance Exercise Scale, ranging from 0, extremely easy to 10 extremely hard [10]. Participants were able to resume training upon completion of testing.

### 2.1. Experimental Procedure

**Figure 1:**
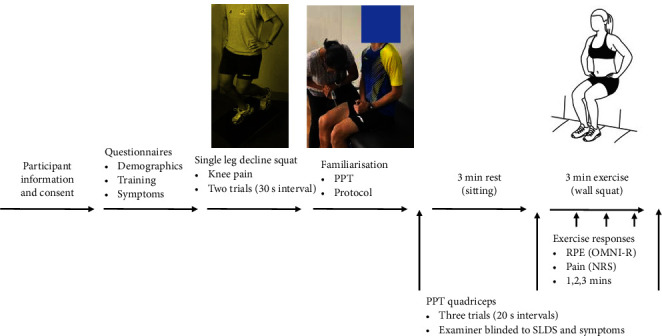
Experimental procedure. PPT: pressure pain threshold; SLDS: single leg decline squat; RPE: rating of perceived exertion; OMNI-R: OMNI-Resistance Exercise Scale; NRS: numerical rating scale.

The experimental procedure is illustrated in Figure 1. Participants first completed questionnaires about demographics, training levels, and history of symptoms over the last 12 months. After this, participants performed a single leg decline squat (SLDS) to record the presence or absence of pain in the test leg (knee with most symptoms or a random leg (coin toss) if symptoms were absent or equal between sides). After this, participants were familiarised with the experimental protocol and measurements before completing the two experimental conditions (rest and exercise) in the same order.

### 2.2. Rest and Exercise Conditions

Participants first completed a resting condition, in which they were asked to sit on the edge of a plinth with their legs hanging freely for 3 minutes. Participants were instructed to “please remain seated with your legs in this position without talking or moving.” Second, they were asked to hold a wall squat position for 3 minutes. During familiarisation, participants were instructed to place their heels on a marker at a heel-to-wall distance of 45 cm for male and 41 cm for female athletes. This distance could be adjusted if needed to achieve the desired 90–100° knee flexion when the thigh is horizontal and the leg vertical. The participant was provided with the following standardised instructions: “When asked, please squat down until your thighs are parallel with the ground and hold this position, without moving for 3 minutes or until you cannot hold it any longer. Immediately after the 3 minutes or upon stopping, please sit on the plinth with your feet relaxed over the edge for your final measures.” The above experimental conditions were based on a published protocol [11].

## 3. Outcome Measures

### 3.1. Exertion and Pain Levels

At each minute of the wall squat, participants were asked to rate their perceived exertion on the OMNI-Resistance Exercise Scale, ranging from 0, extremely easy to 10 extremely hard [10]. They also rated the level of pain in the thigh and anterior knee using 0–10 NRS at each minute during the wall squat exercise. A clipboard displaying the two scales and locations were displayed for visual reference.

### 3.2. Pressure Pain Threshold

PPT was recorded before and after each experimental condition using a digital, hand-held algometer (Somedic AB, Farsta, Sweden) with 1 cm^2^ probe by an examiner blind to symptoms and SLDS responses. The examiner displayed high reliability for PPT measurement in a laboratory environment (intraclass correlation coefficient (ICC) and 95% confidence interval (CI): 0.94 (0.81, 0.98). PPT measured was over quadriceps muscle of the test leg at a point 15 cm from the base of the patella. This location was marked on the skin for consistent application. PPT was measured with the participant seated on the edge of the plinth with their feet hanging freely. Pressure was applied at a rate of 30 kPa/s, until the participant first perceived pain, at which point they pressed a button. The following instructions were provided: “I will apply pressure with this probe to your thigh. The pressure will be gradually increased until you press the button, at which point I will stop. Please press the button the moment you feel the sensation change from one of pressure to that of pressure and pain.” Three PPT trials were performed within a 1 cm radius, with 20 s rest intervals and the mean value used in analysis.

## 4. Baseline Measures

### 4.1. Knee Pain and Symptoms

The Oslo Sports Trauma Research Centre Overuse Injury Questionnaire (OSTRC-O2) was used to record the severity of knee problems in the last 7 days [12]. Items 1 to 4 captured information about participation, training, performance, and pain due to knee problems (in either leg) using 4-point Likert scales. Responses were summed according to previously published methods to provide a total score ranging from 0 (full participation without knee pain) to 100 (unable to participate with severe knee pain) [12, 13]. Participants rated their worst level of knee pain in the test leg in the past 7 days and their worst level of knee pain on the day of testing on a 0–10 NRS, with endpoints of “no pain” and “the worst imaginable pain.”

### 4.2. Single Leg Decline Squat Test

Participants stood on their test leg on a 20° decline board, with their hands on their hips. They were asked to slowly squat to 60° of knee flexion and then return to upright, keeping their trunk upright and heel in contact with the board. This test was repeated twice with a 30 s rest interval. Following each test, participants rated the intensity of any knee pain on a 0–10 NRS and the location(s) of any knee pain using previously used pictorial representation of regions [14, 15]. Participants were divided into two groups based on the presence (NRS ≥ 1) or absence (NRS = 0) of knee pain during this test. The SLDS was chosen as it has been established that one in two elite basketball players [15] and one in four nonelite, adolescent volleyball players experience pain [14].

### 4.3. Statistical Analysis

Reliability of PPT measured in a field-setting was first examined by checking for systematic error between the mean of the three PPT trials collected before and after quiet rest. ICCs and 95% CI were computed using an Excel spreadsheet [16]. The magnitude thresholds for correlations corresponding to 0.99, 0.90, 0.75, 0.50, and 0.20 were described as extremely high, very high, high, moderate, and low reliability. Minimal detectable change (MDC) was computed using the following formulae: MDC = $1.96*\text{SEM}*\sqrt{2}$, where the standard error of measurement (SEM) = $\text{SD}*\sqrt{\left(1-\text{ICC}\right)}$.

Feasibility of the exercise protocol was evaluated as the proportion of participants completing the 3-minute wall squat. Ratings of exertion and thigh pain were considered proxies for the intensity of the exercise stimulus. Absolute differences (PPT after minus PPT before) and relative differences (PPT after minus PPT before, divided by PPT before, multiplied by 100%) were computed as measures of the magnitude of EIH.

The following exploratory analyses were performed using Stata 17.0 (StataCorp, Texas, USA). Data were checked for normality using Shapiro–Wilk tests and is reported as the mean and standard deviation (SD) unless otherwise specified. Exertion and thigh pain during the wall squat were analysed using mixed-model ANOVAs, including time (1, 2, 3 minutes) as a within-subject factor and knee pain during SLDS (yes/no) as a between-subject factor. PPT was analysed using a mixed-model ANOVA, including condition (rest and exercise) and time (before and after) as within-subject factors and knee pain during SLDS (yes/no) as a between-subject factor. *P* values less than 0.05 were considered significant. Bonferroni adjustment was performed by multiplying *p* values by 4 to minimise the chance of error due to multiple comparisons.

## 5. Results

### 5.1. Participant Baseline Characteristics

Fourteen athletes (8 male, 6 female) consented to participate. Seven (50%) of participants reported pain during the SLDS on the test leg and had a mean pain intensity of 3.9 ± 2.0. Symptoms were experienced over the patellar tendon (4 participants, 57%) or patellofemoral joint (2 participants, 29%) and lateral or posterior knee (each 1 participant, 14%). As expected, self-reported measures of knee symptoms (OSTRC-O2, pain in last 7 days and pain on the day of testing) were worse in participants who had knee pain during the SLDS compared with those that were pain-free on this test (Table 1). In contrast, there were no differences in terms of demographic, sporting characteristics, or the intensity of training prior to testing.

### 5.2. Reliability of PPT Measured in a Field-Setting

PPT of the quadriceps muscle did not show any systematic errors between repeated measurements before and after quiet rest (Supplementary Table 1). Very high ICCs were observed for all participants (ICC 0.94 95% CI (0.85, 0.98). ICCs were slightly lower for participants with knee pain (0.91 (0.68, 0.98) versus without knee pain on SLDS (0.97 (0.88, 0.99). MDCs were 116.8 kPa for all participants, 120.6 kPa for participants with knee pain, and 78.3 kPa for participants without knee pain.

### 5.3. Feasibility of the Exercise Protocol

**Figure 2:**
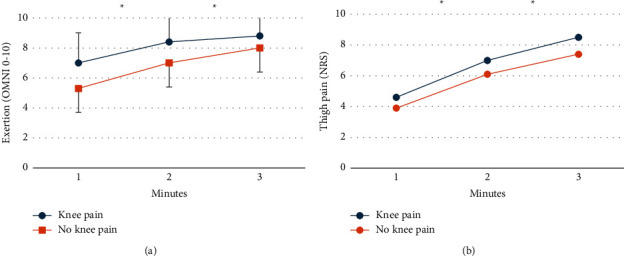
Mean exertion (a) and thigh pain (b) ratings after 1, 2, and 3 minutes of wall squat exercise for each group. Error bars are standard deviations. Significant differences are indicated by asterisks. OMNI-R: OMNI-Resistance Exercise Scale; NRS: numerical rating scale.

Thirteen (93%) participants completed the entire three minutes of wall squat, while one participant completed 2 minutes. Exertion and thigh pain ratings are presented in full in Supplementary Table 2 and illustrated in Figure 2. There were no significant differences in exertion or thigh pain between participants with or without knee pain on SLDS (*p* > 0.16). At 3 minutes of wall squat, mean exertion was 8.4 (1.8), mean thigh pain was 7.9 (2.0), and median knee pain was 0 (interquartile range 1).

### 5.4. Effects of Isometric Exercise and Quiet Rest on PPT

**Figure 3:**
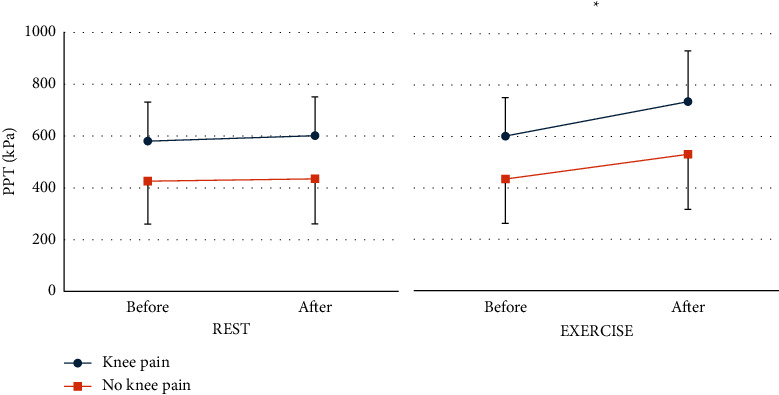
Mean pressure pain threshold (PPT) before and after rest and exercise for participants with and without knee pain on single leg decline squat. Error bars are standard deviations. Significant differences are indicated by asterisks.

PPT data are presented in full in Supplementary Table 3 and illustrated in Figure 3. PPT of the quadriceps muscle increased after exercise but not rest (condition-by-time interaction *p*=0.02). There were no differences in PPT between participants with and without knee pain on SLDS (*p*=0.09). The absolute magnitude of EIH was 115.3 kPa (95% CI 70.5, 160.1) for all participants, which neared the MDC. Absolute differences exceeded the MDC for participants with and without knee pain on SLDS (134.1 kPa 95% CI (4.1, 224.2) and 96.5 kPa (47.9, 145.0), respectively). The relative magnitude of EIH was 22.2% and 22.6% for participants with and without knee pain, respectively.

## 6. Discussion

This study demonstrated that EIH could be feasibly tested in a field setting as evidenced by very high reliability of the test stimulus (PPT of the exercising quadriceps muscles) and successful completion of the exercise stimulus (3-minute wall squat) by 93% of elite athletes, with a mean exertion of 8.4 out of 10. PPT of the exercising quadriceps muscle significantly increased after performing the wall squat (but not after quiet rest), indicating that the exercise task was successful in inducing EIH. The following exploratory finding was also observed. Participants with or without knee pain on the SLDS test showed similar relative increases in PPT (22.2% versus 22.6%), suggesting that the presence of knee pain on the day of testing did not impair EIH response in the current cohort of otherwise healthy athletes. Confirmation of these exploratory findings is necessary in a larger sample of athletes with persistent pain.

Our findings are consistent with previous studies in healthy participants [9, 11, 17]. Vaegter et al. [11] studied young university students, observing a 17% increase in PPTs of the exercising quadriceps muscle after a 3-minute wall squat but not after quiet rest [11]. Their experimental protocol was identical to ours, with the exception that their testing was performed in a laboratory environment with strict pretesting requirements. For example, their participants were advised to refrain from physical exercise, coffee, or nicotine on the day of testing. In the current study, participants were tested during a typical morning training session, with participants reporting a mean training intensity of 3.8 out of 10 prior to testing. Further research is needed to explore potential relationships between the intensity of training and EIH responses.

In contrast to studies of healthy individuals, EIH responses have not been consistently identified after quadriceps exercise in participants with knee conditions [7, 8]. Holden et al. [7] studied isometric and dynamic resistance exercise of the quadriceps muscles in participants with patellar tendinopathy [7], finding no increase in PPT locally over the patellar tendon following exercise. Similarly, in young females with chronic patellofemoral pain, Straszek et al. [8] observed no increase in PPT over the patella after dynamic resistance exercise of the quadriceps muscles [8]. However, in both studies, there was a significant increase in PPT at the tibialis anterior muscle, suggesting that the location of the test stimulus may influence findings. In the current study, testing was only performed over the exercising quadriceps muscle, which may explain the similar magnitude of EIH between participants with and without knee pain. Our rationale for testing over muscle was that previous reviews demonstrate that the magnitude of EIH is greatest over exercising muscles [18]. Future studies may wish to measure PPT over both the exercising muscle and the patellar tendon, as the latter may provide information reflecting the net balance between ascending pain facilitatory and descending pain inhibitory pathways. We also recommend the 3-minute wall squat task as it induced minimal knee pain while strong activation of muscle nociceptors, which is thought to be important in triggering endogenous descending inhibitory pathways [17].

Testing of EIH in athletes may be of clinical importance as impaired endogenous pain inhibitory function may impact performance, rehabilitation, or risk of chronic pain. If such testing is to have clinical utility, it is important to first understand potential sources of variability in EIH responses between and within participants. Previous investigation of the magnitude of EIH found poor reliability when healthy participants were tested on two occasions separated by one week [11]. The authors speculated that greater variability may occur in clinical pain populations. This may be true, as in the current study, reliability estimates were lower and MDC values were higher for participants with knee pain, although they were still very high for both groups. Future research may wish to monitor within-participant changes in endogenous pain inhibitory function over the course of a season.

A strength of this study was that it followed an established laboratory protocol and included a quiet rest condition. Another strength was that one examiner tested for the presence of pain during the SLDS, while another examiner performed PPT measures blind to this information. The main weakness of the study is the small sample size; hence, all findings are considered preliminary and require confirmation. It is also recognised that participants in the current study did not undergo a clinical examination and likely experienced a range of knee conditions. Hence, it cannot be discounted that other samples of athletes with specific knee conditions and/or reporting persistent difficulty participating in sport may show different findings.

## 7. Conclusions

This study demonstrated that EIH was feasible to assess courtside in athletes during an in-season training session using measures previously adopted in a laboratory environment. Following a 3-minute isometric wall squat, elite badminton athletes displayed increased PPT of the exercising quadriceps muscle (hypoalgesia) indicating descending pain inhibition. Athletes with and without knee pain displayed similar changes in PPT, providing preliminary evidence that EIH may not be impacted by knee pain in otherwise healthy athletes. Recommendations for future testing of athletes are proposed.

## Figures and Tables

**Table 1:** Characteristics of all participants and participants who had knee pain during the single leg decline squat compared to those that were pain-free on this test.

Demographic/sport	All participants	Knee pain	No knee pain	*P* value
*n*	14	7	7	
Male *n* (%)	8 (57%)	5 (71%)	3 (43%)	0.59
Female *n* (%)	6 (43%)	2 (29%)	4 (57%)	
Age (years)	24.4 (5.5)	24.4 (7.0)	24.6 (4.1)	0.89
Height (cm)	170.6 (6.9)	169.6 (7.1)	171.7 (7.1)	0.58
Weight (kg)	66.7 (9.5)	69.9 (10.1)	63.6 (8.6)	0.23
Sport experience (years)	13.8 (5.9)	13.3 (6.9)	14.3 (5.1)	0.76
Sport (hours/week)	10.3 (5.0)	10.7 (4.8)	9.9 (5.6)	0.78
Training intensity prior to testing (OMNI-R 0–10)	3.8 (1.3)	4.4 (1.5)	3.3 (0.8)	0.17

Knee symptoms				

Knee pain last 7 days (NRS)	2.4 (2.1)	4.4 (3.3)	0.4 (0.8)	0.009^∗^
Knee pain today (NRS)	1.4 (2.2)	2.7 (2.4)	0 (0)	0.01^∗^
OSTRC-O2 (0–100)	12.4 (19.2)	24.7 (21.1)	0 (0)	0.009^∗^

Data represent count (%) or mean (SD). OMNI-R: OMNI-Resistance Exercise Scale; NRS: numerical rating scale; OSTRC-O2: Oslo Sports Trauma Research Centre Overuse Injury Questionnaire and Significant differences between Knee pain and No knee pain groups are highlighted by an asterisk.

## Data Availability

Data are available from Dr. Brooke Coombes b.coombes@griffith.edu.au for researchers who meet the criteria for access to confidential data.

## Supplementary Materials

Supplementary Materials Supplementary Table 1. Test-retest reliability for PPT collected before and after quiet rest. Supplementary Table 2. Exertion and thigh pain during each minute of the wall squat for all participants and for participants with and without knee pain on the single leg decline squat. Supplementary Table 3. Pressure pain threshold (PPT) for all participants and for participants with and without knee pain on the single leg decline squat.
