# Supplementary material for: Is It Time to Step outside the Laboratory? The Feasibility of Field-Based Examination of Exercise-Induced Hypoalgesia in Elite Badminton Athletes with and without Knee Pain
Source: Transl Sports Med. 2024 Jun 11;2024:2953220. doi: 10.1155/2024/2953220 (PMC11221980; doi:10.1155/2024/2953220)
Supplement: Supplementary Materials — Supplementary Table 1. Test-retest reliability for PPT collected before and after quiet rest. Supplementary Table 2. Exertion and thigh pain during each minute of the wall squat for all participants and for participants with and without knee pain on the single leg decline squat. Supplementary Table 3. Pressure pain threshold (PPT) for all participants and for participants with and without knee pain on the single leg decline squat. [file 2953220.f1.docx]

**Supplementary Table 1.** Test-retest reliability for PPT collected before and after quiet rest. ICC: Intraclass correlation coefficients, SEM: standard error of measurement, MDC: minimal detectable change.

|  | ICC (95% CI) | SEM (kPa) | MDC (kPa) |
| --- | --- | --- | --- |
| All participants | 0.94(0.85, 0.98) | 42.1 | 116.8 |
| Knee pain | 0.91 (0.68,0.98) | 43.5 | 120.6 |
| No knee pain | 0.97 (0.88, 0.99) | 28.2 | 78.3 |

**Supplementary Table 2.** Exertion and thigh pain during each minute of the wall squat for all participants and for participants with and without knee pain on the single leg decline squat. Data represents mean (SD). OMNI-R: OMNI-Resistance Exercise Scale; NRS: numerical rating scale

|  | **1 min** | **2 min** | **3 min** |
| --- | --- | --- | --- |
| **Exertion (OMNI-R 0-10)** |  |  |  |
| All participants | 6.1 (2.1) | 7.7 (2.0) | 8.4 (1.8) |
| Knee pain | 7.0 (2.0) | 8.4 (2.1) | 8.8 (2.0) |
| No knee pain | 5.3 (1.9) | 7.0 (1.6) | 8.0 (1.6) |
| **Thigh pain (NRS 0-10)** |  |  |  |
| All participants | 4.2 (2.4) | 6.6 (1.9) | 7.9 (2.0) |
| Knee pain | 4.6 (2.9) | 7.0 (1.8) | 8.5 (1.9) |
| No knee pain | 3.9 (1.9) | 6.1 (2.0) | 7.4 (2.1) |

**Supplementary Table 3.** Pressure pain threshold (PPT) for all participants and for participants with and without knee pain on the single leg decline squat. P-values are reported unadjusted and adjusted for repeated comparison. Absolute differences (PPT after minus PPT before) and relative differences (PPT after minus PPT before, divided by PPT before, multiplied by 100%) are reported as mean differences and 95% confidence intervals (CI).

|  | **Before**  **rest**  **(kPa)** | **After**  **rest**  **(kPa)** | **Unadj**  **P-value** | **Bonf adj**  **P-value** | **Absolute difference**  **(95% CI) (kPa)** | **Relative difference**  **(95% CI) (%)** |
| --- | --- | --- | --- | --- | --- | --- |
| All participants | 503.0 (172.2) | 518.0 (177.9) | 0.42 | 1.00 | 15.0 (-23.7, 53.6) | 3.3 (-6.0, 12.6) |
| Knee pain | 580.4 (150.6) | 601.6 (149.5) | 0.51 | 1.00 | 21.1 (-53.0, 95.3) | 4.6 (-8.7, 18.0) |
| No knee pain | 425.7 (166.1) | 434.4 (173.0) | 0.70 | 1.00 | 8.8 (-43.6, 61.2) | 2.0 (-15.2, 19.3) |
|  | **Before exercise (kPa)** | **After**  **exercise**  **(kPa)** | **Unadj**  **P-value** | **Bonf adj**  **P-value** | **Absolute difference**  **(95% CI) (kPa)** | **Relative difference**  **(95% CI) (%)** |
| All participants | 518.0 (177.9) | 633.3 (224.4) | <0.001 | <0.001 | 115.3 (70.5, 160.1) | 22.4 (15.1, 29.7) |
| Knee pain | 601.6 (149.5) | 735.7 (197.2) | 0.01 | 0.04 | 134.1 (44.1, 224.2) | 22.3 (6.4, 38.1) |
| No knee pain | 434.4 (173.0) | 530.9 (213.9) | 0.003 | 0.012 | 96.5 (47.9, 145.0) | 22.6 (16.1, 29.1) |
